# A Deep Learning System for Fully Automated Retinal Vessel Measurement in High Throughput Image Analysis

**DOI:** 10.3389/fcvm.2022.823436

**Published:** 2022-03-22

**Authors:** Danli Shi, Zhihong Lin, Wei Wang, Zachary Tan, Xianwen Shang, Xueli Zhang, Wei Meng, Zongyuan Ge, Mingguang He

**Affiliations:** ^1^State Key Laboratory of Ophthalmology, Zhongshan Ophthalmic Center, Sun Yat-sen University, Guangzhou, China; ^2^Faculty of Engineering, Monash University, Melbourne, VIC, Australia; ^3^Centre for Eye Research Australia, East Melbourne, VIC, Australia; ^4^Department of Ophthalmology, Guangdong Provincial People's Hospital, Guangdong Eye Institute, Guangdong Academy of Medical Sciences, Guangzhou, China; ^5^Guangzhou Vision Tech Medical Technology Co., Ltd., Guangzhou, China; ^6^Research Center and Faculty of Engineering, Monash University, Melbourne, VIC, Australia

**Keywords:** artificial intelligence, automated analysis, hierarchical vessel morphology, cardiovascular disease, epidemiology

## Abstract

**Motivation:**

Retinal microvasculature is a unique window for predicting and monitoring major cardiovascular diseases, but high throughput tools based on deep learning for in-detail retinal vessel analysis are lacking. As such, we aim to develop and validate an artificial intelligence system (Retina-based Microvascular Health Assessment System, RMHAS) for fully automated vessel segmentation and quantification of the retinal microvasculature.

**Results:**

RMHAS achieved good segmentation accuracy across datasets with diverse eye conditions and image resolutions, having AUCs of 0.91, 0.88, 0.95, 0.93, 0.97, 0.95, 0.94 for artery segmentation and 0.92, 0.90, 0.96, 0.95, 0.97, 0.95, 0.96 for vein segmentation on the AV-WIDE, AVRDB, HRF, IOSTAR, LES-AV, RITE, and our internal datasets. Agreement and repeatability analysis supported the robustness of the algorithm. For vessel analysis in quantity, less than 2 s were needed to complete all required analysis.

## Introduction

The morphology of the retinal vessels is closely correlated with the microvascular state of the body. The retinal vasculature is organized within a delicate, optimized structure that minimizes shear stresses due to blood flow and energy used for perfusion, achieving sufficient energy supply with minimal cost (1). Changes in retinal vascular morphology have previously been reported to be associated with a wide range of ocular and systemic diseases (2–5), including life-threatening cardiovascular disease. Deviation from the geometric ideal and measurement of vessel changes may provide a quantitative assessment of vessel deformity and pathology. Quantification of these changes may enhance our understanding of the relationship between ocular and systemic changes and promote the use of the retinal vessels as novel biomarkers in the management of chronic diseases.

Computer-assisted technology has enabled the quantification of retinal morphology. A series of machine learning methods and software tools have been developed for the quantified assessment of the retinal vasculature. Widespread use of these tools however has been limited due to their need for manual input [IVAN (6), SIVA (7), VAMPIRE (8)], time-consuming nature [IVAN (6), SIVA (7)], applicability to only specific retinal regions [IVAN (6), SIVA (7)], or a limited number of measurement parameters [IVAN (6), VAMPIRE (8), QUARTZ (9, 10)].

Deep learning (DL) has been established in recent years as the dominant paradigm for retinal image processing. It has outperformed other machine learning (ML) methods in achieving retinal vessel segmentation with minimal time and state-of-the-art accuracy (11). Widespread adoption in real-world settings however depends on its ability to address variations in image quality and artifacts, resolutions and modality of various fundus cameras, and the interference of pathologic lesions on vessel segmentation. A further common challenge for vessel segmentation is broken vessels at branching or crossing points, which often result in misclassification of arteries and veins, or discontinued vessels. In addition to vessel segmentation, SIVA-DLS (12) is a recently developed deep learning system that directly predicts vessel caliber based on cropped retinal fundus without performing segmentation. However, this tool is restricted to a limited region of the retina and evaluates only a small number of vessel parameters.

Training deep learning algorithms with larger datasets and sufficient variation may help address these challenges. However, given the labor intensiveness in labeling vessels manually, there are much fewer training data available for vessel segmentation than disease classification. Most databases with annotated vessels used in algorithm development are homogeneous, small, and free of eye diseases, compromising the adoption of algorithms trained on these data in real-world clinical settings.

As such, we developed and validated a deep learning system (Retina-based Microvascular Health Assessment System, RMHAS) using multi-source data to provide fast, reliable, and detailed retinal vessel quantification. We intend to provide RMHAS as a public tool to enable automated high-throughput retinal vessel analysis on large collections of fundus images.

## Methods

### Study Design and Overview

**Figure 1:**
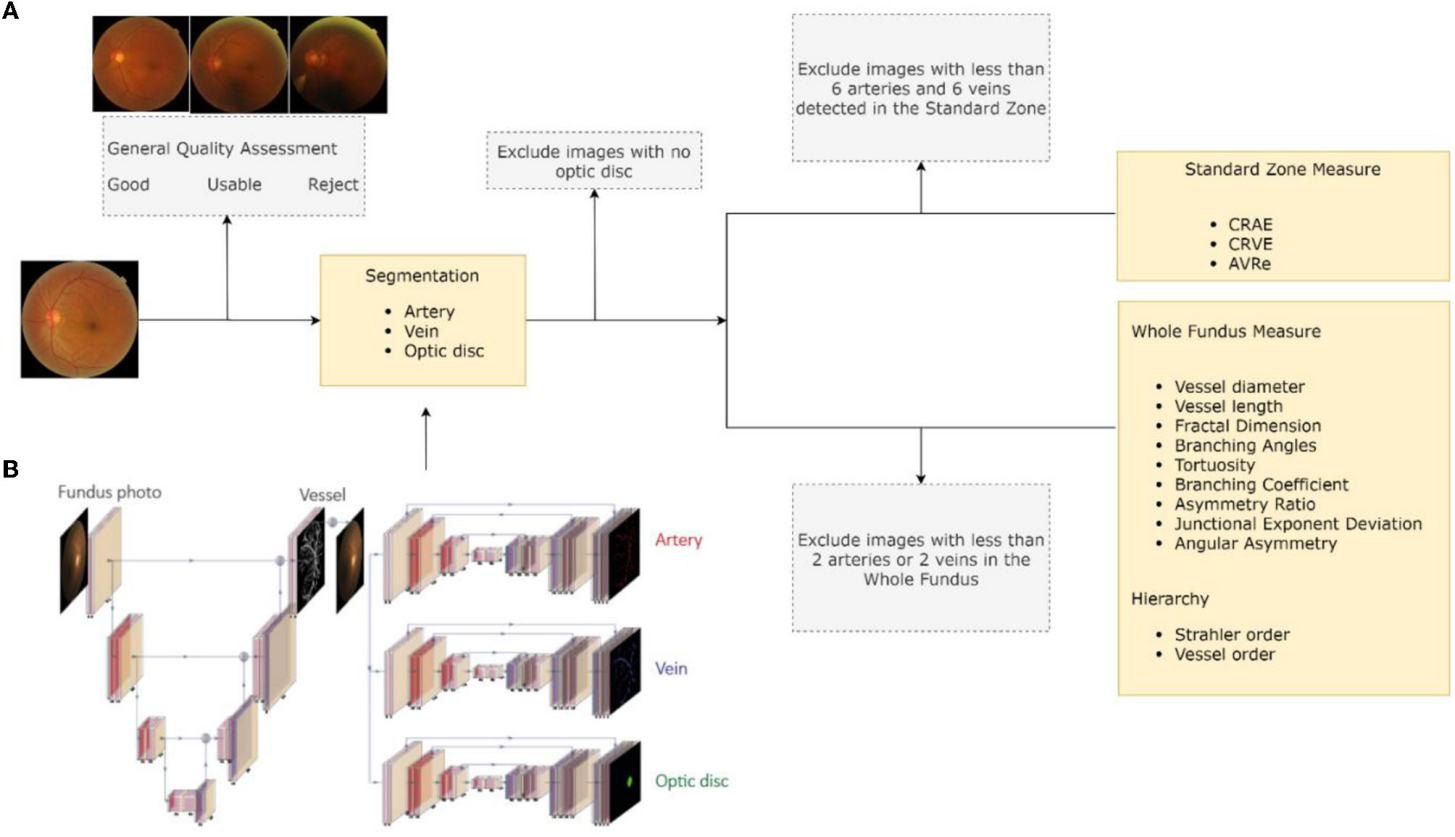
Software development flowchart. **(A)** Retina-based Microvascular Health Assessment System (RMHAS) workflow. **(B)** The multi-branch U-Net used in RMHAS for segmentation. The trunk generates an intermediate retinal vessel feature map, which is concatenated with the input image and divided into three separate branches for retinal artery, vein, and optic disc segmentation.

RMHAS consisted of several functional parts. Firstly, the image quality assessment module assessed overall image quality before segmentation. Secondly, the segmentation module generated artery, vein, and optic disc segmentation maps. Thirdly, based on segmentation, the measurement module computed region-specific measurements within the Standard zone (a zone 0.5–1.0 disc diameter away from the optic disk margin), (13) and global physical or geometric measures for the whole fundus image. Lastly, a second quality assessment was carried out to filter out abnormal measurements and exclude incompetent detections based on specific criteria. Final results were subsequently generated. Figure 1A outlines a flowchart of the software development process.

### Fundus Datasets

#### In-house Dataset

To train the segmentation algorithm for RMHAS, we built a large manually labeled retinal artery/vein segmentation database. This database included diverse eye diseases, age groups, and imaging devices. Two hundred and twenty images with significant variations were initially included, of which 20 came from the UK Biobank (14), 120 from the LabelMe platform (15). 50 from the Lingtou Eye Cohort Study (LECS) (16). and 30 from the Guangzhou Twin Eye Study (GTES) (17). These images were composed of 60 images with diabetic retinopathy ranging from mild to severe non-proliferative, 20 images with age-related macular degeneration (AMD), 20 with glaucoma, 20 with pathologic myopia (PM), and 20 with hypertension. 20 images for each age group of <18, 18 to 50, and ≥ 50 years were included. To represent healthy images, another 200 images were randomly selected from the UK Biobank population-based study (Table 1).

**Table 1:** The composition of the newly-built dataset for retinal artery, vein, and optic disc segmentation. LECS, Lingtou Eye Cohort Study; GTES, Guangzhou Twin Eye Study.

**Category**	**Number of images**		**Source**
DR		60	LabelMe
r1	20		
r2	20		
r3	20		
Glaucoma		20	LabelMe
AMD		20	LabelMe
PM		20	LabelMe
HBP		20	LECS
Age		60	
<18	20		GTES
18–50	20		GTES, LECS
50+	20		LECS
UK Biobank		20	UK Biobank
UK Biobank		200	UK Biobank
**Total**		**420**	

Vessel annotation was performed according to the following procedure. Firstly, we generated artery/vein (A/V) segmentation maps by supplying the fundus images to the W-Net model (18). After this, image graders using custom software can modify or fine-tune vessel segmentation as per A/V segmentation maps. Secondly, to make low-contrast small vessels more identifiable, we carried out image enhancements using contrast limited adaptive histogram equalization (CLAHE). Pre-segmentations generated by the W-Net model could be overlaid on the original fundus or the augmented image. Image graders could switch between different modalities to verify their segmentation and erase or add vessels. The custom software interface developed for this image annotation process is illustrated in Supplementary Figure 1.

All images were randomly assigned to one of four image graders who were trained by an ophthalmologist. These image graders were requested to independently modify and segment the retinal arteries, veins, and optic disc. To assess inter-observer variability, ~10 percent of images were repeatedly labeled between different graders. To assess intra-observer consistency, around 10 percent of the images were repeatedly labeled by the same grader. Cohen's kappa score was computed to assess inter-observer and intra-observer reliability.

#### Public Datasets

To improve model generalizability and robustness, data from 20 public datasets were also used in the development of the segmentation algorithm. The STARE (19), VEVIO (20, 21). CHASEDB (22), DR HAGIS (23), UoA-DR (24), and PRIME-FP20 (25) datasets were used for vessel segmentation. The RITE (26), HRF (27), AV-WIDE (28, 29), IOSTAR (30, 31), LES-AV (32), and AVRDB (33) datasets were used for artery and vein classification. For optic disc segmentation, the ONHSD (34), DRIONS-DB (35), Drishti-GS (36), RIGA (37), REFUGE (38), G1020 (39), PALM (40), and ADAM (41) datasets were used. Although those datasets have previously been used to develop segmentation algorithms, their label quality varies.

In summary, a diverse collection of datasets composed of fundus images of varying image qualities, resolutions, pathologies, and modalities were included in developing this algorithm. As the size of datasets varied significantly, training and validation set splits were carried out as follows: 20 official training and test set images were split from the CHASEDB dataset; if the dataset had fewer than 100 total images, training and test images followed an 80/20 split; and if the dataset had more than 100 images, only 20 images were split into the test set. Table 2 describes the characteristics of the 21 datasets used to develop the segmentation algorithm. Diagrams in Supplementary Figure 2 outlines the train/test division.

**Table 2:** Characteristics of the 21 datasets used to develop the segmentation algorithm. Only images with available labels were included. AMD, age-related macular degeneration; HR, hypertensive retinopathy; PM, pathologic myopia; DR, diabetic retinopathy.

**Dataset**	**Label**	**Year**	**No**.	**Centered**	**Field**	**Size**	**Eye disease**	**Camera**
STARE	vessel	2000	20	macula	30°-45°	605 ×700	various	TRV-50 fundus camera (Topcon)
VEVIO	vessel	2011	16	macula		640 ×480 600 ×500		Video indirect ophthalmoscopy
CHASEDB	vessel	2012	28	optic-disc	25°	960 ×999	–	NM-200D (Nidek, Japan)
DR HAGIS	vessel	2017	40	macula	45°	2816 ×1,880 4,752 ×3,168	DR, HBP, AMD, glaucoma	TRC-NW6s (Topcon), TRC-NW8 (Topcon), or CR-DGi (Canon)
UoA-DR	vessel, optic disc	2017	200	macula-disc	45°	–	DR	–
PRIME-FP20	vessel	2020	15	macula	200°	4,000 ×4,000	DR	Optos 200Tx (Optos plc, Dunfermline, Scotland, UK)
RITE	artery/vein	2013	40	macula	45°	565 ×584	DR	CR5 non-mydriatic 3CCD camera (Canon)
HRF	artery/vein, optic disc	2013	45	macula	45°	3,504 ×2,336	DR, glaucoma	
AV-WIDE	artery/vein	2015	30	macula	200°	1,300 ×800 2,816 ×1,880 1,500 ×900	DR	Optos 200Tx (Optos plc, Dunfermline, Scotland, UK)
IOSTAR	artery/vein, optic disc	2015	30	macula	45°	1,024 ×1,024		SLO (i-Optics Inc., the Netherlands)
LES-AV	artery/vein	2018	22	optic-disc	30°−45°	1,620 ×1,444 1,958 ×2,196	glaucoma	
AVRDB	artery/vein	2020	100	macula-disc	30°	1,504 ×1,000	HR, DR	
ONHSD	optic disc	2004	99	macula	45°	640 ×480	DR	CR6 45MNf fundus camera (Canon)
DRIONS-DB	optic disc	2008	110	optic-disc	30°	600 ×400	glaucoma, ocular hypertension	
Drishti-GS	optic disc	2014	50	macula	25°	2,045 ×1,752	glaucoma	–
RIGA dataset	optic disc	2018	750	macula-disc	–	2,240 ×1,488 2,743 ×1,936 2,376 ×1,584	DR, glaucoma	–
REFUGE2	optic disc	2020	1200	macula		2,124 ×2,056 1,634 ×1,634	glaucoma	Zeiss Visucam 500/Canon CR-2
G1020	optic disc	2020	1020	macula-disc	45°	–	various	–
PALM	optic disc	2019	400	macula-disc	–	–	PM	–
ADAM	optic disc	2020	400	macula	–	–	AMD	–
Ours	artery/vein, optic disc	2021	420	macula-disc	various	various	DR, glaucoma, AMD, PM	Various

The study was conducted in accordance with the Declaration of Helsinki, using deidentified retinal photographs from previously published studies. Ethics Committee ruled that approval was not required for this study.

### Image Quality Assessment

As noted earlier, the first functional part of RMHAS is a classification of overall image quality before before vessel segmentation. This was carried out using a convolutional neural network (CNN) model built from the EyeQ dataset (42), and enabled classification of overall image quality into three grades: “good,” “usable,” and “reject.” Images with clear and identifiable main structures and lesions, but with some low-quality factors (blur, insufficient illumination, shadows) were classified as “usable”. Images with serious quality issues that could not be reliably diagnosed by an ophthalmologist were classified as “reject”.

A second quality assessment was performed after segmentation. Images with the following conditions were excluded: no detectable optic disc; <6 arteries six veins detectable in the Standard zone; or <2 arteries and two veins detected in the whole fundus. Excluded images, the reason for their exclusion, and their available measurements were saved separately from the main measurements.

### Optic Disc and Vessel Segmentation

We extended the U-Net component from W-Net (18) into multiple branches to enable simultaneous and efficient retinal artery, vein, and optic disc segmentation. Figure 1B outlines the details of the RMHAS segmentation architecture. The input for RMHAS was a fundus image, cropped to the field of view (FOV) and resized to 512 ×512 pixels. The first intermediate layer generated a segmentation map based on the whole retinal vessel map and concatenated it to the original fundus image. This first segmentation map could then be used by the downstream network as an attention map, to focus more on targeted areas of the image. The following segmentations were carried out in three separate branches for the retinal arteries, veins, and optic disc, using these features as guidance.

We trained the RMHAS step-by-step by first training the root branch to generate an intact vessel map. The root branch was then frozen, with the artery, vein, and optic branches unfrozen and trained iteratively. We trained RMHAS with a preset of 200 epochs, a batch size of 8, and a cosine-shaped learning rate from 0.1 to 0.00001. To tackle class imbalance issues – i.e., far more background pixels than foreground (vessel) pixels, we used a weighted combination of Cross-entropy loss and Dice loss (1:3) as the objective function (detailed in the Supplementary Methods). The Adam optimizer (43) was used in backpropagation to minimize the objective function by optimizing the model parameters. To reduce overfitting, we did data augmentation by random horizontal and vertical flipping, rotating between 0 and 45°, and by transforming contrast and illumination (Supplementary Methods). We also used early stopping if validation loss did not improve for 10 epochs. To alleviate issues of broken vessels at branch-ing/crossing points, we performed further data augmentation by specifically cropping out a random number of branching/crossing regions with random sizes for training (to create more pieces of crossing vessel segments and increase variations). This model was trained on the PyTorch platform.

### Retinal Vessel Measurement

We measured retinal vessel morphology by using custom region-specific summarization and global physical/geometric parameters. For region-specific summarization, the vessel calibers were summarized as central retinal artery equivalent (CRAE) and central retinal vein equivalent (CRVE) from the 6 largest arteries and veins detected in the Standard zone, based on the revised Knudtson-Parr-Hubbard formula (44). Artery to vein ratio from equivalents (AVRe) was generated by dividing CRAE by CRVE. For global physical/geometric parameters, vessels were converted into segments separated by interruptions at the branching or crossing points. Short vessels <10 pixels in length were excluded from the analysis. Using methods similar to SIVA (13), the diameters (mean, standard deviation [SD]), arc length, chord length, length diameter ratio (LDR), tortuosity, branching angle (BA), branching angle from edges (BA_edge), branching coefficient (BC), angular asymmetry (AA), asymmetry ratio (AR), junctional exponent deviation (JED) were measured and computed. The vessel orders and Strahler orders of each segment were built using graphical representation, resulting in a series of hierarchical nodes and edges. In summary, 16 basic parameters were included. Detailed formulas and methods are presented in the Supplementary Methods. Graphs were built using the Python package NetworkX.

### Accuracy of Segmentation

We assessed the accuracy of segmentation at the pixel level.

Quantitative evaluation criteria including the area under the receiver operator characteristic curve (AUC), accuracy, sensitivity, specificity, between manually labeled and predicted segmentations were computed.

Qualitative evaluation was performed by overlaying predicted segmentations with manually labeled segmentation, using different colors for visual analysis.

For external validation, we performed retinal vessel segmentation and width measure using the pubic REVIEW (45) dataset as reference.

### Reliability of Vessel Measurements

For reliability, intraclass correlation coefficient (ICC) and Bland–Altman plots were used to assess agreement in Standard zone measurements between manually labeled and predicted segmentation.

For repeatability, the ICCs were computed between all measurements on photographs taken repeatedly under similar illumination and locations for the same eye with the same camera.

ICC values of <0.5, 0.5–0.75, 0.75–0.9, and ≥ 0.90 are indicative of poor, moderate, good, and excellent reliability, respectively (46).

Statistical analysis was completed with R version 4.0.1 and Python 3.6.

### Data Availability Statement

The UK Biobank is an open-access resource to researchers through registration of proposed research. The remaining in-house dataset is available from the corresponding author upon reasonable request.

### Code Availability Statement

The code of this study is available from the corresponding author upon request. All models were built using publicly available software and packages.

## Results

**Figure 2:**
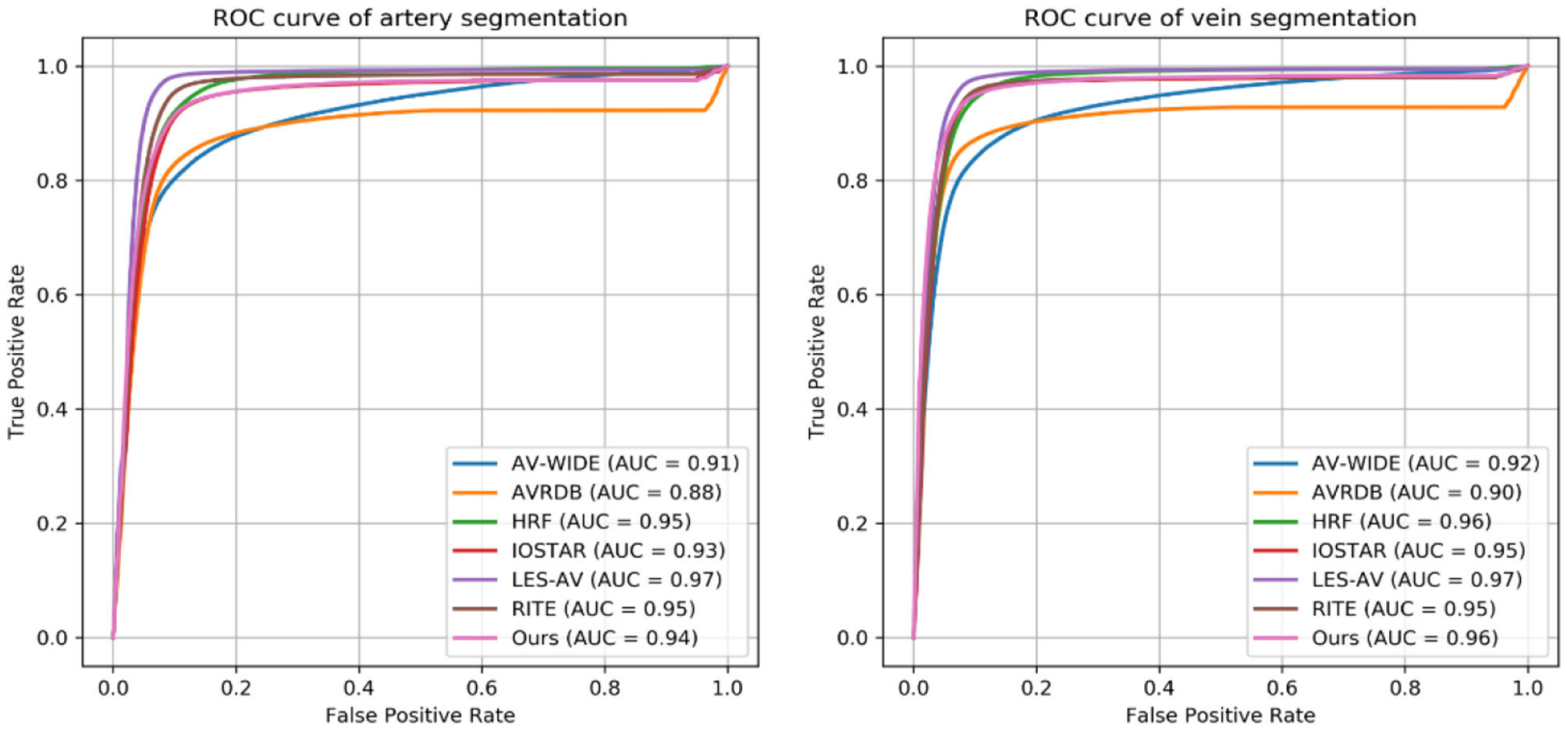
Receiver operating characteristic (ROC) curves of Retina-based Microvascular Health Assessment System (RMHAS) for segmentation of artery and vein within different datasets.

**Figure 3:**
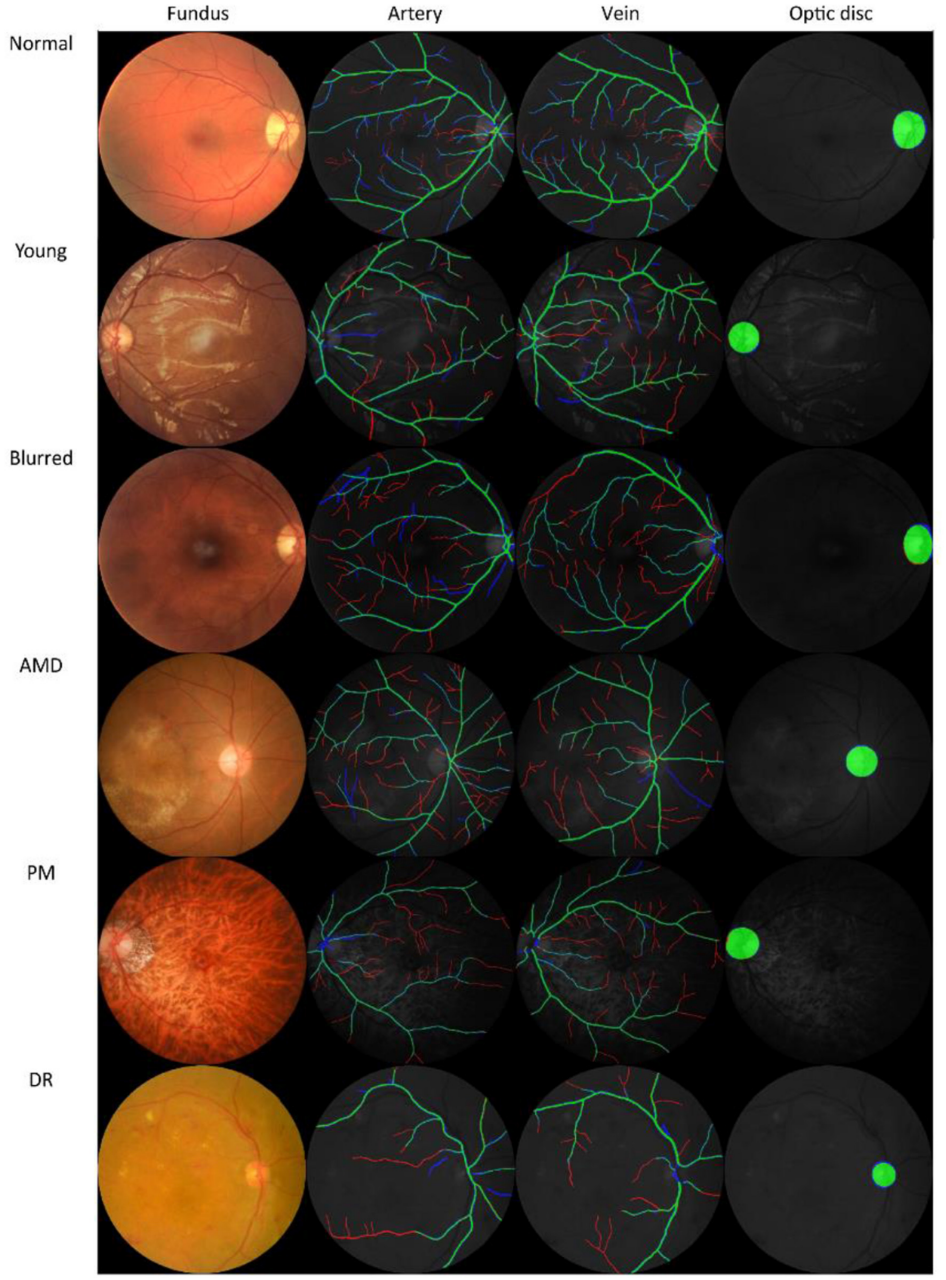
Examples of model prediction by Retina-based Microvascular Health Assessment System (RMHAS) versus manual segmentation. Blue pixels: false negatives (pixels that were manually labeled but missed by the model). Red pixels: false positives (pixels identified by the model but missed by manual labeling). Green pixels: pixels with consistent segmentation between model and manual labeling. AMD, age-related macular degeneration; PM, pathologic myopia; DR, diabetic retinopathy.

The four observers achieved moderate consistency in intra- and inter-observer agreement analysis. Detailed kappa scores are presented in Supplementary Table 1. For segmentation accuracy, the algorithm achieved AUC (95% CI) of 0.914 (0.914–0.915), 0.913 (0.913–0.914), 0.948 (0.948–0.948), 0.919 (0.918–0.920), 0.959 (0.959–0.960), 0.953 (0.952–0.953), 0.922 (0.922–0.922) for artery segmentation and 0.930 (0.929–0.931), 0.940 (0.939–0.940), 0.956 (0.956–0.956), 0.935 (0.934–0.936), 0.961 (0.961–0.962), 0.959 (0.959–0.960), 0.948 (0.948–0.949) for vein segmentation on the AV-WIDE, AVRDB, HRF, IOSTAR, LES-AV, RITE and our dataset, respectively. Figure 2 plots the model's ROC curves in different datasets. Detailed evaluation results are presented in Table 3. Figure 3 shows representative examples of overlaid segmentations for images with different features, including a normal fundus, fundus image from young participants with prominent retinal nerve fiber layer reflections, blurred image from older participants, fundus with AMD, PM, and severe DR. Blue pixels represent false negatives (pixels that were manually labeled but missed by the model). Red pixels represent false positives (pixels identified by the model but missed by manual labeling). Green pixels represent pixels with consistent segmentation between model and manual labeling.

**Table 3:** Segmentation performance of Retina-based Microvascular Health Assessment System (RMHAS) on the test set in different datasets.

	**Accuracy**		**Sensitivity**		**Specificity**		**F1 score**	
	**Artery**	**Vein**	**Artery**	**Vein**	**Artery**	**Vein**	**Artery**	**Vein**
AV-WIDE	0.95	0.95	0.68	0.73	0.96	0.96	0.45	0.47
AVRDB	0.94	0.95	0.72	0.78	0.95	0.96	0.47	0.62
HRF	0.93	0.94	0.83	0.87	0.93	0.94	0.46	0.50
IOSTAR	0.94	0.95	0.72	0.77	0.95	0.96	0.51	0.59
LES-AV	0.95	0.95	0.86	0.85	0.96	0.96	0.58	0.61
RITE	0.94	0.94	0.86	0.87	0.94	0.95	0.57	0.63
Ours	0.95	0.96	0.72	0.80	0.96	0.97	0.48	0.57

For external validation, the vessel segmentation performance and width measure results in the REVIEW database are shown in the Supplementary Table 2 and Supplementary Figure 3.

Agreement between retinal vessel caliber in the Standard zone, measured on RMHAS predicted segmentation and human segmentations were estimated using ICC. Agreements of vessel equivalent measurements on our dataset were excellent, good, or excellent on LES-AV (a dataset composed purely of disc-centered fundus images) but moderate or below on AVRDB, HRF, RITE (composed of macula-centered photos), suggesting to achieve ideal Standard zone measures, images should be optic-disc centered (similar to SIVA). Detailed ICC results are presented in Table 4. Bland-Altman plots of the agreement of (a) retinal arteriolar caliber and (b) retinal venular caliber, (c) AVRe between manual and predicted vessel maps, (d) differences between AVRe measures on manual and predicted vessel maps; vs. the distance of the optic disc center to the edge of FOV are displayed in Supplementary Figure 3.

**Table 4:** Agreement estimates of retinal-vessel caliber in the Standard zone for measurements on RMHAS segmentation and manual segmentation.

	**ICC (95%CI)**	
**Dataset**	**CRAE**	**CRVE**
AVRDB (*n* = 54)	0.55 (0.37–0.69)*	0.30 (0.08–0.49)
HRF (*n* = 38)	0.59 (0.38–0.74)*	0.42 (0.18–0.62)
LES-AV (*n* = 20)	0.89 (0.78–0.95)**	0.90 (0.80–0.95)***
Ours (*n* = 238)	0.93 (0.91–0.94)***	0.97 (0.96–0.97)***
RITE (*n* = 34)	0.43 (0.17–0.64)	0.55 (0.31–0.72)*

*Moderate: between 0.5 and 0.75, **Good: between 0.75 and 0.9, ***Excellent: >0.90. ICC, intraclass correlation; CI, confidence interval; CRAE, central retinal artery equivalent; CRVE, central retinal vein equivalent; n, the number of images.

The reproducibility and robustness of the measurements were measured by comparing measurements generated from photographs taken repeatedly under similar conditions. For measurements in the Standard zone, 198 of 1290 (15.3%) images failed quality control in Standard zone measures. Of these, 9, 22, and 120 images were classified as good, usable, and reject in the first quality assessment module. The model achieved excellent agreement for measurements generated under similar conditions (Table 5A). For measurements within the whole fundus, 8 (0.6%) of images failed quality control in whole fundus measures and generated 114,809 vessel segments for analysis. The model achieved moderate to good agreement for measurements based on all vessels (Table 5B).

**Table 5A:** Agreement estimates of measurements in the Standard zone on photographs taken repeatedly under similar conditions.

**Location**	**Quality**	**ICC (95%CI)**	
		**CRAE**	**CRVE**
Disc centered	Good (*n* = 67)	0.89 (0.84–0.93)**	0.92 (0.88–0.95)***
	Reject (*n* = 129)	0.78 (0.71–0.83)**	0.83 (0.78–0.87)**
	Usable (*n* = 14)	0.98 (0.94–0.99)***	0.94 (0.86–0.98)***
Macula centered	Good (*n* = 264)	0.94 (0.93–0.95)***	0.95 (0.94–0.96)***
	Reject (*n* = 43)	0.78(0.65–0.86)**	0.81(0.70–0.88)**
	Usable (*n* = 29)	0.93 (0.88–0.96)***	0.91 (0.84–0.95)***

*Moderate: between 0.5 and 0.75, **Good: between 0.75 and 0.9, ***Excellent: >0.90. ICC, intraclass correlation; CI, confidence interval; CRAE, central retinal artery equivalent; CRVE, central retinal vein equivalent.

**Table 5B:** Agreement estimates of measurements based on the whole fundus on photographs taken repeatedly under similar conditions.

	**Good**	**Usable**	**Reject**
**Artery**	***n*** **=** **330**	***n*** **=** **35**	***n*** **=** **161**
Arc	0.75 (0.70–0.79)*	0.55 (0.32–0.72)*	0.56 (0.46–0.64)*
Chord	0.76 (0.71–0.79)**	0.54 (0.31–0.71)*	0.56 (0.46–0.64)*
Length diameter ratio	0.80 (0.76–0.83)**	0.64 (0.44–0.78)*	0.59 (0.50–0.67)*
Mean diameter	0.78 (0.74–0.81)**	0.74 (0.58–0.85)*	0.65 (0.57–0.72)*
Weighted diameter	0.81 (0.78–0.84)**	0.80 (0.68-0.88)**	0.74 (0.68–0.80)*
**Vein**	***n*** **=** **330**	***n*** **=** **34**	***n*** **=** **137**
Arc	0.75 (0.71–0.79)**	0.68 (0.49–0.80)*	0.58 (0.48–0.66)*
Chord	0.76 (0.72–0.79)**	0.66 (0.46–0.79)*	0.59 (0.49–0.67)*
Length diameter ratio	0.78 (0.74–0.81)**	0.76 (0.60–0.86)**	0.63 (0.53–0.70)*
Mean diameter	0.80 (0.76–0.83)**	0.61 (0.40–0.76)*	0.73(0.66–0.79)*
Weighted diameter	0.82 (0.78–0.85)**	0.69 (0.51–0.82)*	0.74 (0.66–0.79)*

*Moderate: between 0.5 and 0.75, **Good: between 0.75 and 0.9, ***Excellent: >0.90. ICC, intraclass correlation; CI, confidence interval; CRAE, central retinal artery equivalent; CRVE, central retinal vein equivalent; Weighted diameter: mean diameter weighted by segment length.

**Figure 4:**
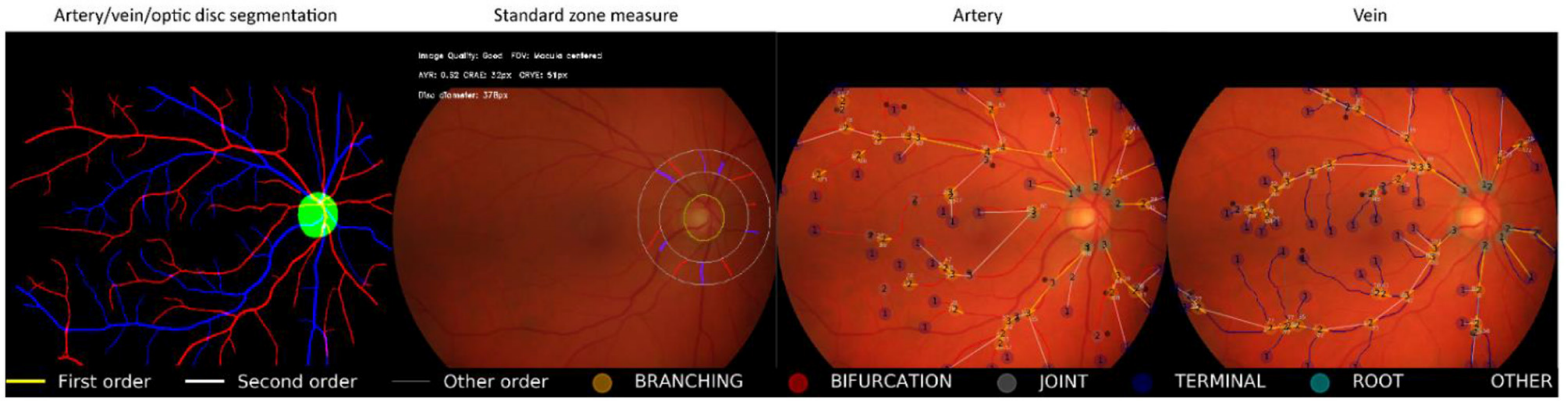
Illustration of Retina-based Microvascular Health Assessment System (RMHAS) output. From left to right: artery, vein, and optic disc segmentation; parameters measured in the standard zone; parameters measured in the whole fundus for artery and vein, respectively. Measures are demonstrated and plotted visually. Users can examine the performance of each functional part throughout the analysis.

Figure 4 shows an example of the RMHAS model output. Measures are demonstrated and plotted visually. Users can easily evaluate the performance of each functional part throughout the analysis.

## Discussion

### Algorithm Development

Retinal vessel segmentation is challenging and often compromised by interference from the central light reflex, image quality variation and artifact, poor image contrast of small vessels, broken vessels at branching/crossing points, and pathological retinal lesions.

To tackle these challenges, we first built the largest manually labeled retinal artery and vein segmentation dataset known to date, to train the segmentation algorithm. Secondly, we specifically designed a deep learning architecture that harnessed a two-stage sequential segmentation, where the intermediate vessel segmentation was used to guide subsequent multi-branch segmentations. The separate branches that segmented arteries, veins, and the optic disc reduced the difficulty in distinguishing artery and vein pixels from a single branch. Thirdly, we carried out data augmentation specifically for artery and vein crossing areas.

### Functionality

RMHAS addresses the limitations of existing algorithms and software, including IVAN (6), SIVA (7), and VAMPIRE (8). which are semi-automatic and have limited regions of interest (ROI). IVAN (6) and SIVA (7) require more than 20 min to process each image, and QUARTZ (9) takes on average 53.6 s per analysis. The QUARTZ (9) platform can analyze whole fundus images but has few output parameters (artery/vein width and tortuosity). SIVA-DLS (12) is the only published deep learning system to use fundus images and predict vessel caliber end-to-end without vessel segmentation. It was built based on measured CRAE, CRVE values from SIVA. This method is straightforward but might lack interpretability. Further, SIVA-DLS is restricted to examining the Extended zone (from 0.5 to 2.0-disc diameter) (13) and has limited output parameters (CRAE, CRVE, AVR only).

In comparison, the RMHAS algorithm provides a far larger number of physical and geometric parameters without sacrificing efficiency. In addition to standard vessel caliber measurements, RMHAS provides measurements on tortuosity, LDR, JED, AR with additional topological information. These measurements are unitless and are less sensitive to diametric measurement noise. The Strahler order corresponds to branching complexity (47). Vessel order describes the conventional order of division of each branch of a vessel. These measurements facilitate flexibility in subsequent analysis. For example, they could be stratified when summarizing the thickness or length of a vessel; or calculated as a global representation of the overall or specified retinal vascular network.

### Accuracy of Segmentation

AUC scores were high across different datasets, achieving high pixel-level segmentation accuracy. Interestingly, the visualization of overlaid manual-predicted segmentation suggested that model predictions outperformed manual labeling, especially for small vessels that human graders often missed. For challenging cases, including images from young participants with highly reflective retinal nerve fiber layers, elderly participants with blurred retinal images, or retinal images with existing eye diseases, the algorithm provided segmentations more accurate than human graders.

### Validity and Repeatability of Vessel Measurement

Internal validation demonstrated reproducibility and robustness in vessel measurements. In general, vessel calibers measured within the Standard zone in disc-centered images were most robust. All measurements achieved good or better agreement.

For external validation, we should note that measurements are often not directly comparable between different algorithms or software, particularly for measurements with units. For example, CRAE and CRVE measurements between SIVA and other software tools have been previously reported as not equivalent, despite these caliber measurements being associated with the same systemic health risk factors (48). Discrepancy in caliber measurement is often due to variation in magnification during image acquisition. A meaningful comparison would require Littmann's method (49) to adjust the magnification factor by considering refractive error, corneal curvature, and axial length and adapting them for Gullstrand's schematic eye. More importantly, this adjustment method would require fundus cameras to be constructed based on a telecentric ray path. However, most fundus cameras currently on the market do not strictly follow this principl (50). Further, even when magnification is appropriately adjusted, caliber should ideally be measured when the structure of interest is in the same position within the photo, although this is virtually impossible. All of these factors may result in variations in the caliber measurement (50). Given these challenges and the need to enable measures on images with unknown fundus camera and magnification settings, we chose to present caliber measurements in pixel units rather than micron values. Notably in the Bland-Altmann plots (Supplementary Figure 3), the variance in differences increases as retinal vessel caliber increases for both venules and arterioles in our dataset, which we assume was resulted from the diversity of the dataset, which is constituted of images from different cameras. However, their ratios were more stable. This suggests when analyzing images across different cameras, relevant measurements should be adjusted, for example, CRAE be adjusted by CRAE, or use the ratio values instead.

### Efficiency and Potential for Future Adoption

Table 6 summarizes and compares existing retinal vessel measurement algorithms and software. RMHAS achieved sufficient reliability and efficiency in critical retinal vessel measurements. With 558,420 parameters, the algorithm required <2 s to complete all the segmentation and analysis when running images in batches on a server with one GeForce GTX TITAN GPU (Nvidia Inc., CA, USA) and an Intel Core i7-4790K CPU. The mean time cost for each task within the algorithm, as tested by analyzing 100 images, were as follows: image quality: 0.02 s; artery, vein, optic disc segmentation: 0.05 s; Standard zone measure: 0.17 s; vessel graph building: 0.51 s; graph plotting: 1 s.

**Table 6:** Comparison of different algorithms and software for retinal vessel analysis.

	**IVAN**	**SIVA**	**VAMPIRE**	**QUARTZ**	**SIVA-DLS**	**RMHAS (ours)**
Processing time	20 min	25 min	-	53.57s	A few seconds	<2 s
Kind	Semi-automatic	Semi-automatic	Semi-automatic	Automatic	Automatic	Automatic
ROI	Standard	Standard + Extended	Whole fundus	Whole fundus	Standard + Extended	Standard + Whole fundus
Algorism	ML	ML	ML	ML	DL	DL
AVR	√	√	√	√	√	√
Mean vessel diameter	√	√	√	√	√	√
Length-diameter ratio	×	√	×	√	×	√
Vessel tortuosity	×	√	√	√	×	√
Branching coefficients	×	√	√	×	×	√
Branching angle	×	√	√	√	×	√
Angular asymmetry	×	√	×	×	×	√
Asymmetry ratio	×	√	×	×	×	√
Junctional exponent deviation	×	√	×	×	×	√
Fractal dimension	×	√	√	×	×	√
Hierarchical vessel tree	×	×	×	×	×	√
Year	2004	2010	2011	2015	2020	2021

*ROI, region of interest; AVR, artery to vein ratio*.

RMHAS has several strengths. It is fast, fully automatic, interpretable, easily accessible, and provides a wide range of measurement parameters with orders. It can handle challenging images, including retinal images with DR, AMD, glaucoma, or images collected from the very young or elderly. Finally, the algorithm is compatible with images obtained from various fundus cameras with different image resolutions. RMHAS limitations include its measurement of retinal caliber value based on pixel units rather than micron measures due to unknown image magnification factors.

## Conclusion

RMHAS achieved good segmentation accuracy across datasets with diverse eye conditions and image resolutions. Compared with manual segmentation, RMHAS performed better at outlining small vessels than human graders, especially in challenging cases. The agreement and repeatability analysis supported the robustness of the algorithm. RMHAS was feasible for application in automated high throughput retinal vessel analysis and required minimal time. We intend to provide RMHAS as a public tool for the research community. The algorithm demo is publicly available (https://www.retinavessel.com/) for testing and analysis. For batch analysis in large quantities, please contact us.

## Data Availability Statement

The raw data supporting the conclusions of this article will be made available by the authors, without undue reservation.

## Author Contributions

MH conceptualized and designed the study and had full access to all data. DS and ZL did the deep learning modeling. DS did the literature search and wrote the first draft of the manuscript. DS, WW, and XS did the statistical analysis. ZT, WW, XZ, ZG, and MH reviewed and modified the manuscript. All authors commented on the manuscript.

## Funding

This work was supported by Fundamental Research Funds of the State Key Laboratory of Ophthalmology, National Natural Science Foundation of China (82171075), Science and Technology Program of Guangzhou, China (202002020049), and Project of Special Research on Cardiovascular Diseases (2020XXG007). MH receives support from the University of Melbourne Research Accelerator Program and the CERA Foundation. The Center for Eye Research Australia receives Operational Infrastructure Support from the Victorian State Government. The sponsor or funding organization had no role in the design or conduct of this research. The sponsor or funding organization had no role in the design, conduct, analysis, or reporting of this study. The funding sources did not participate in the design and conduct of the study, collection, management, analysis interpretation of the data, preparation, review, or approval of the manuscript, and decision to submit the manuscript for publication.

## Conflict of Interest

WM was employed by Guangzhou Vision Tech Medical Technology Co., Ltd. The remaining authors declare that the research was conducted in the absence of any commercial or financial relationships that could be construed as a potential conflict of interest.

## Publisher's Note

All claims expressed in this article are solely those of the authors and do not necessarily represent those of their affiliated organizations, or those of the publisher, the editors and the reviewers. Any product that may be evaluated in this article, or claim that may be made by its manufacturer, is not guaranteed or endorsed by the publisher.

## Abbreviations

RMHAS: retina-based microvascular health assessment system
AMD: age-related macular degeneration
PM: pathologic myopia
DR: diabetic retinopathy
HR: hypertensive retinopathy
LECS: lingtou eye cohort study
GTES: guangzhou twin eye study
CLAHE: contrast limited adaptive histogram equalization
CNN: convolutional neural network
FOV: field of view
CRAE: central retinal artery equivalent
CRVE: central retinal vein equivalent
AVRe: artery to vein ratio from equivalents
LDR: length diameter ratio
BA: branching angle
BA_edge: branching angle from edges
BC: branching coefficient
AA: angular asymmetry
AR: asymmetry ratio
JED: Junctional exponent deviation
SD: standard deviation
CI: confidence interval
ICC: intraclass correlation coefficient
ROC: receiver operator characteristic
AUC: area under the receiver operator characteristic curve
ROI: region of interest.
