# Supplementary material for: A Deep Learning System for Fully Automated Retinal Vessel Measurement in High Throughput Image Analysis
Source: Front Cardiovasc Med. 2022 Mar 22;9:823436. doi: 10.3389/fcvm.2022.823436 (PMC8980780; doi:10.3389/fcvm.2022.823436)

**Contents**

**Methods**

**1. Data augmentation**

*1.1 General augmentation:* We used the Torch vision library for data augmentation (<https://pytorch.org/vision/stable/_modules/torchvision/transforms/>), detailed settings:

RandomHorizontalFlip()

RandomVerticalFlip()

RandomRotation(degrees=45, fill=(0, 0, 0), fill_tg=0)

RandomAffine(degrees=0, scale=(0.95, 1.20))

RandomAffine(degrees=0, translate=(0.05, 0))

RandomChoice([scale, transl, rotate])

brightness, contrast, saturation, hue = 0.25, 0.25, 0.25, 0.01

ColorJitter(brightness, contrast, saturation, hue)

*1.2 Crossing points augmentation:*

1. Performs vessel skeletonization by Zhang’s algorithm[1]. 2.Detect the vessel crossing points by template kernels following method offered by Jean-Pat https://quabr.com/16241708/hit-and-miss-transform-for-detecting-branched-point-and-endpoint-in-scikit-image. 3. Randomly select 5 - 20 crossing points, draw circle or bounding boxes centered at them with a diameter generated by the original image height devided by a factor randomly chosen from 18-22. 3. Crop the circles or bounding boxes (mask other region) for training.

Source Target Skeletonization Crossing points


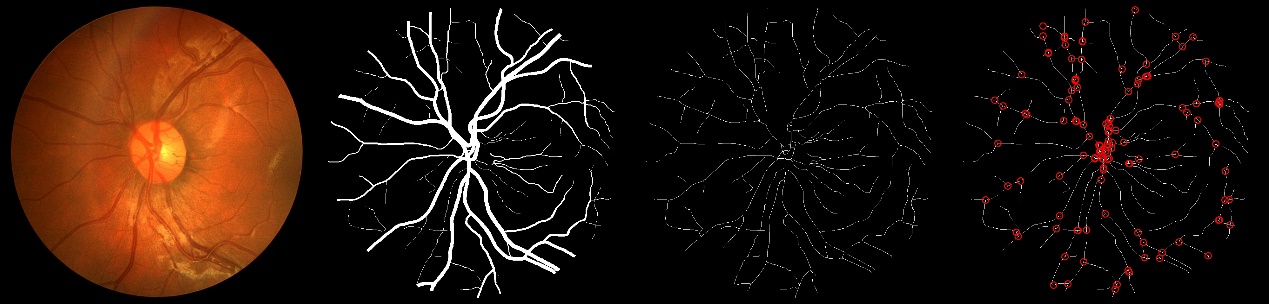


Source augmentation Target augmentation Source augmentation Target augmentation


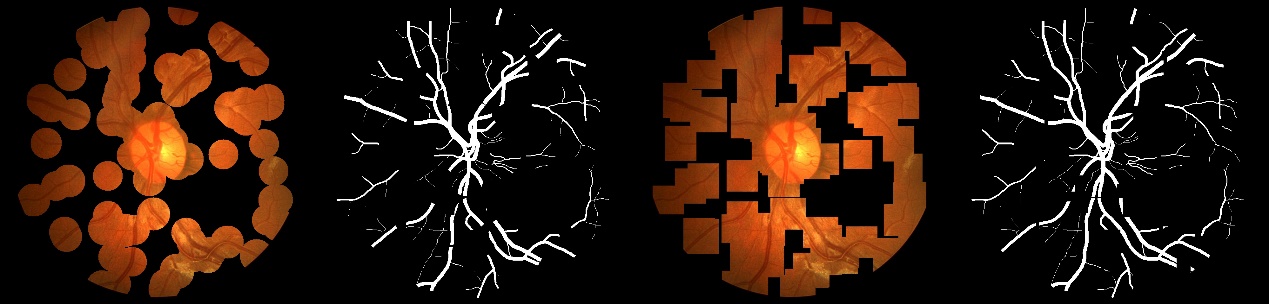


**2. Loss function**

Cross entropy (CE), the most commonly used loss function, was used for image segmentation. This loss examines each pixel individually, comparing the class predictions vector to the one-hot encoded target (or ground truth) vector. For binary segmentation, CE can be defined as:

$$\mathrm{CE}(p,\hat{p})=-(p\log(\hat{p})+(1-p)\log(1-\hat{p}))$$

Where $p\in\{0,1{\}}^{n}\text{ and }0\leq\hat{p}\leq1. p\text{ and }\hat{p}$ are the ground truth and predicted segmentation.

Dice loss (DL), is another popular loss function for image segmentation. DL is based on the Dice coefficient, which is a measure of overlap between two samples and is equivalent to the F1 score. The measure ranges from 0 to 1, where a value of 1 denotes perfect overlap. The Dice coefficient (DC) is calculated as:

$$\mathrm{DC}=\frac{2| p\cap\hat{p}|}{|p|+|\hat{p}|}$$

Dice loss can directly optimize the Dice coefficient, with no requirement for class re-weighting on imbalanced data, can be defined as: 1 - DC

**3. Vessel tracing and graph building**

After removing the optic disc region, vessels from the binary vessel map were skeletonized. Vessel trees were sorted by the index of connected components. Vessel tracing started from the points closest to the optic disc on the vessel skeleton, points with more than 4 connections were classified as bifurcations, points with 3 connections were classified as branching points.

Directed graphs were built by adding edges to NetworkX object, with directions starting from points closer to the optic disc**.**[2]

**4.Vessel Measurements**[3,4]

**4.1 Region-specific Measurements**

Measured in Standard Zone, the ring area of 0.5 to 1.0 disk diameter from the optic disc.

*Central Retinal Artery/Vein Equivalent*

According to the revised Knudtson–Parr–Hubbard formula[5] , the six largest arteries and veins in the Standard Zone are summarized as the central retinal artery/vein equivalent (CRAE/CRVE). A central vessel equivalent is obtained by iteratively pairing up the largest vessels with the smallest and repeating until a single value is reached. The six largest arteries and veins are referred to as the big 6.

$$\begin{aligned} \mathbf{&}\text{ Arteries: }\hat{\mathbf{W}}\boldsymbol{=0.88 \times}\sqrt{\left( \mathbf{w}_{\mathbf{1}}^{\mathbf{2}}\mathbf{+}\mathbf{w}_{\mathbf{2}}^{\mathbf{2}} \right)} \\ \mathbf{&}\text{ Veins: }\hat{\mathbf{W}}\boldsymbol{=0.95 \times}\sqrt{\left( \mathbf{w}_{\mathbf{1}}^{\mathbf{2}}\mathbf{+}\mathbf{w}_{\mathbf{2}}^{\mathbf{2}} \right)} \end{aligned}$$

where w1, w2 is a pair of width values and $\hat{\mathbf{W}}$ denotes the new combined width value for the next iteration.

*Arteriovenous Ratio from Equivalents (AVRe)*

Arteriovenous Ratio is calculated from CRAE/CRVE within the standard zone.

**4.2 Global Physical/Geometric Measurements**

After binarization of the segmentation map, distance transform is performed on the binary mask, which gives the Euclidean distance of each vessel pixel from its closest non-vessel pixel. Vessel widths (diameters) are obtained by retrieving the distance value along the vessel centerline. Vessels are converted into segments separated by interruptions at the branching or crossing points.

*Mean Diameter ω(s)*

Mean diameter of a vessel segment(s) is the average value of the segment vessel widths.

*Standard Deviation σ(s)*

The standard deviation of a vessel segment(s)

*Length λ(s)*

The length of s is calculated by the sum of the pairwise Euclidean distance between two adjacent points on the centerline of s, in other words, the arc of the centerline of the segment.

*Length Diameter Ratio: LDR(s)*

The ratio of the length of the segment*(s)* to its average diameter

*Tortuosity τ(s)*

Simple tortuosity is calculated as the arc–chord ratio, τ(s) = λ(s)/C(s) where λ(s) is the length of s, C is the chord of s, measured by the Euclidean distance between the first and last points of the centerline of s.

Curve tortuosity is calculated by the mean segment angles between each branch sampled at a length of 10 pixels.

*Branching Coefficient: BC*

$$\mathbf{BC=}\frac{\boldsymbol{\omega}\left( \mathbf{s}_{\mathbf{1}} \right)^{\mathbf{2}}\boldsymbol{+\omega}\left( \mathbf{s}_{\mathbf{2}} \right)^{\mathbf{2}}}{\boldsymbol{\omega(s}\mathbf{)}^{\mathbf{2}}}$$

where s is the root segment of vessel v and s1 and s2 are daughters of s

*Asymmetry Ratio: AR*

$$\mathbf{AR=}\left( \frac{\mathbf{min}\left\{ \boldsymbol{\omega}\left( \mathbf{s}_{\mathbf{1}} \right)\boldsymbol{,\omega}\left( \mathbf{s}_{\mathbf{2}} \right) \right\}}{\mathbf{max}\left\{ \boldsymbol{\omega}\left( \mathbf{s}_{\mathbf{1}} \right)\boldsymbol{,\omega}\left( \mathbf{s}_{\mathbf{2}} \right) \right\}} \right)^{\mathbf{2}}$$

s1 and s2 denote the daughters of the root segment of v

*Junctional Exponent Deviation: JED(v)*

The junctional exponent deviation is the deviation from the theoretical optimal value of 3 from Murray's law.

$$\boldsymbol{JED=}\frac{\sqrt[\boldsymbol{3}]{\boldsymbol{\omega(s}\boldsymbol{)}^{\boldsymbol{3}}\boldsymbol{-\omega}\left( \boldsymbol{s}_{\boldsymbol{1}} \right)^{\boldsymbol{3}}\boldsymbol{-\omega}\left( \boldsymbol{s}_{\boldsymbol{2}} \right)^{\boldsymbol{3}}}}{\boldsymbol{\omega(s)}}$$

where s1 and s2 are the daughters of the root segment s of v

*Branching Angle: BA*

The branching angle of a vessel is computed by measuring the angles between the sampled centerline (10 pixels) of the root segment s of v and its daughter segments s1 and s2 near the branching point.

*Branching Angle from edges: BA_edge*

The branching angle from edges is computed by measuring the angles between the graph edges of the root segment s of v and its daughter segments s1 and s2.

*Angular Asymmetry: AA*

Angular asymmetry of a vessel measures the absolute difference between the angles of each daughter vessel with the root vessel.

$$\mathbf{AA}\boldsymbol{= |\theta1 - \theta2|}$$

*Fractal Dimension: FD*

Fractal dimension is calculated by the box-counting method[6], the region of interest is covered with rectangular box-like grid with boxes with side length of ε, where different scales ε are being used, expressed as the number of pixels. N (ε) corresponds to the number of boxes needed to completely cover the fractal-like structure at a given scale ε.

$$\boldsymbol{FD}=\lim_{\boldsymbol{c}\boldsymbol{\to0}} \frac{\mathbf{log}\boldsymbol{N}\mathbf{(}\boldsymbol{\varepsilon}\mathbf{)}}{\mathbf{-log}\boldsymbol{\varepsilon}}$$

Fractal dimension of total vessel (FDt), fractal dimension of arteries (FDa), fractal dimension of veins (FDv) are calculated respectively.

**Orders of Measurements**

*Vessel order:*

Developed based on the principle that the starting vessel is termed first order and each subsequent branch has a higher order. If a segment has a diameter more than 3/4 of its parent segment, the order remained the same with the parent. Third order and beyond were categorized as “other”.

*Node kind:*

Terminal node: connected to only one neighbor branch.

Root node: the closest point on a vessel center line to the optic disc. Treated as the seed point in vessel tracking.

Bifurcation node: the junction of two daughter branches with same vessel order.

Branching node: the junction of two daughter branches with different vessel orders.

*Strahler order:*

Strahler number is a numerical measure of branching complexity and the corresponding Strahler order represents a given hierarchical level. The Strahler order is computed as follows: the terminal node of a vessel and the corresponding segment are of level 1. The hierarchy of the subsequent nodes and edges is built in an endocentric way: the Strahler order of the daughter branch after a junction is calculated by adding the maximum Strahler order of the two parent branches and adding an additional 1 if two parents have different orders.[7]

**Reference:**

1. Zhang, T.Y.; Suen, C.Y. A Fast Parallel Algorithm for Image Processing and Computer Vision Robert M. Haralick Thinning Digital Patterns Editor. **1984**, *27*, 4.

2. DiGraph—Directed Graphs with Self Loops — NetworkX 2.6.2 Documentation Available online: https://networkx.org/documentation/stable/reference/classes/digraph.html (accessed on 6 February 2022).

3. Patton, N.; Aslam, T.M.; MacGillivray, T.; Deary, I.J.; Dhillon, B.; Eikelboom, R.H.; Yogesan, K.; Constable, I.J. Retinal Image Analysis: Concepts, Applications and Potential. *Progress in Retinal and Eye Research* **2006**, *25*, 99–127, doi:10.1016/j.preteyeres.2005.07.001.

4. Ng, E.Y.K.; Acharya, U.R.; Suri, J.S.; Campilho, A. Image Analysis and Modeling in Ophthalmology. 402.

5. Knudtson, M.D.; Lee, K.E.; Hubbard, L.D.; Wong, T.Y.; Klein, R.; Klein, B.E.K. Revised Formulas for Summarizing Retinal Vessel Diameters. *Current Eye Research* **2003**, *27*, 143–149, doi:10.1076/ceyr.27.3.143.16049.

6. Liebovitch, L.S.; Toth, T. A Fast Algorithm to Determine Fractal Dimensions by Box Counting. *Physics Letters A* **1989**, *141*, 386–390, doi:10.1016/0375-9601(89)90854-2.

7. Reeb, C.; Kaandorp, J.; Jansson, F.; Puillandre, N.; Dubuisson, J.-Y.; Cornette, R.; Jabbour, F.; Coudert, Y.; Patiño, J.; Flot, J.-F.; et al. Quantification of Complex Modular Architecture in Plants. *New Phytologist* **2018**, *218*, 859–872, doi:https://doi.org/10.1111/nph.15045.

**Table 1.** Intra- and inter-observer agreement analysis in the manually labeled dataset.

|  | | Intra-observer agreement | | | | Inter-observer agreement | | | |
| --- | --- | --- | --- | --- | --- | --- | --- | --- | --- |
|  | Observer | | n | Kappa, Mean (SD) | | Observer | n | Kappa, Mean (SD) | |
| Artery | rater1 | | 12 | 0.63 | (0.11) | rater1-rater4 | 4 | 0.72 | (0.07) |
|  | rater2 | | 5 | 0.59 | (0.09) | rater2-rater4 | 24 | 0.66 | (0.05) |
|  | rater3 | | 5 | 0.60 | (0.06) | rater3-rater1 | 18 | 0.63 | (0.06) |
|  | rater4 | | 5 | 0.60 | (0.04) |  |  |  |  |
| Vein | rater1 | | 12 | 0.69 | (0.08) | rater1-rater4 | 4 | 0.75 | (0.02) |
|  | rater2 | | 5 | 0.65 | (0.05) | rater2-rater4 | 24 | 0.72 | (0.04) |
|  | rater3 | | 5 | 0.68 | (0.05) | rater3-rater1 | 18 | 0.68 | (0.04) |
|  | rater4 | | 5 | 0.65 | (0.02) |  |  |  |  |

Kappa values indicate levels of agreement: 0.40-0.59 weak; 0.60-0.79 moderate, 0.80-0.90 strong, 0.90+ almost perfect.

**Table 2.** Vessel width measurement of Retina-based Microvascular Health Assessment System (RMHAS) on REVIEW dataset. CLRIS (Central Light Reflex Image Set), HRIS (High Resolution Image Set), VDIS (Vascular Disease Image Set), SD (standard deviation).

|  | RHMAS measurement | | | Observer 1 (O1) | | Observer 2 (O2) | | Observer 3 (O3) | |
| --- | --- | --- | --- | --- | --- | --- | --- | --- | --- |
| Dataset | Success rate | Mean | SD | Mean | SD | Mean | SD | Mean | SD |
| CLRIS | 100% | 13.53 | 3.80 | 13.19 | 4.01 | 13.68 | 4.22 | 14.52 | 4.25 |
| HRIS | 100% | 4.73 | 1.36 | 4.12 | 1.25 | 4.35 | 1.35 | 4.58 | 1.26 |
| VDIS | 100% | 7.60 | 2.52 | 8.50 | 2.54 | 8.91 | 2.69 | 9.15 | 2.67 |

**Figure 1.** Vessel labeling user interface. Raw model predictions were overlaid on corresponding fundus images to assist manual modification/segmentation. By switching between the original fundus and CLAHE image, observers can better distinguish small vessels with low contrast. CLAHE: Contrast Limited Adaptive Histogram Equalization.


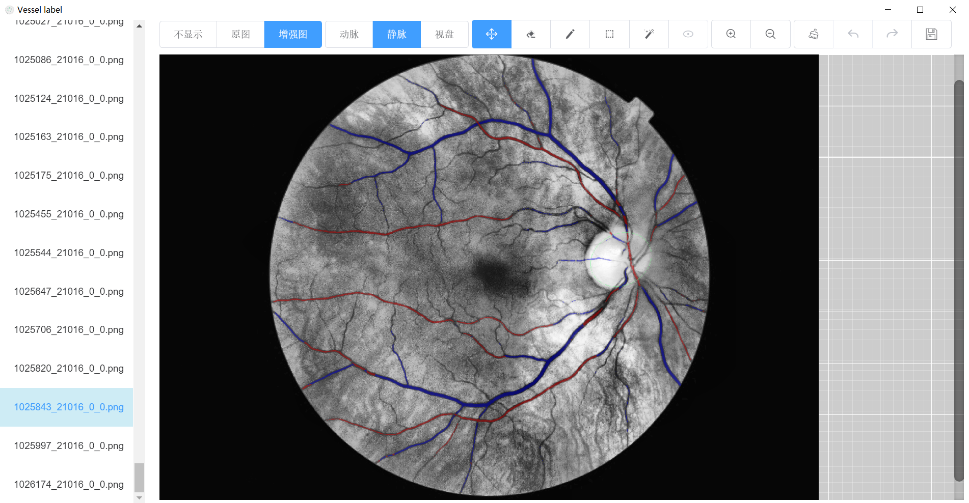

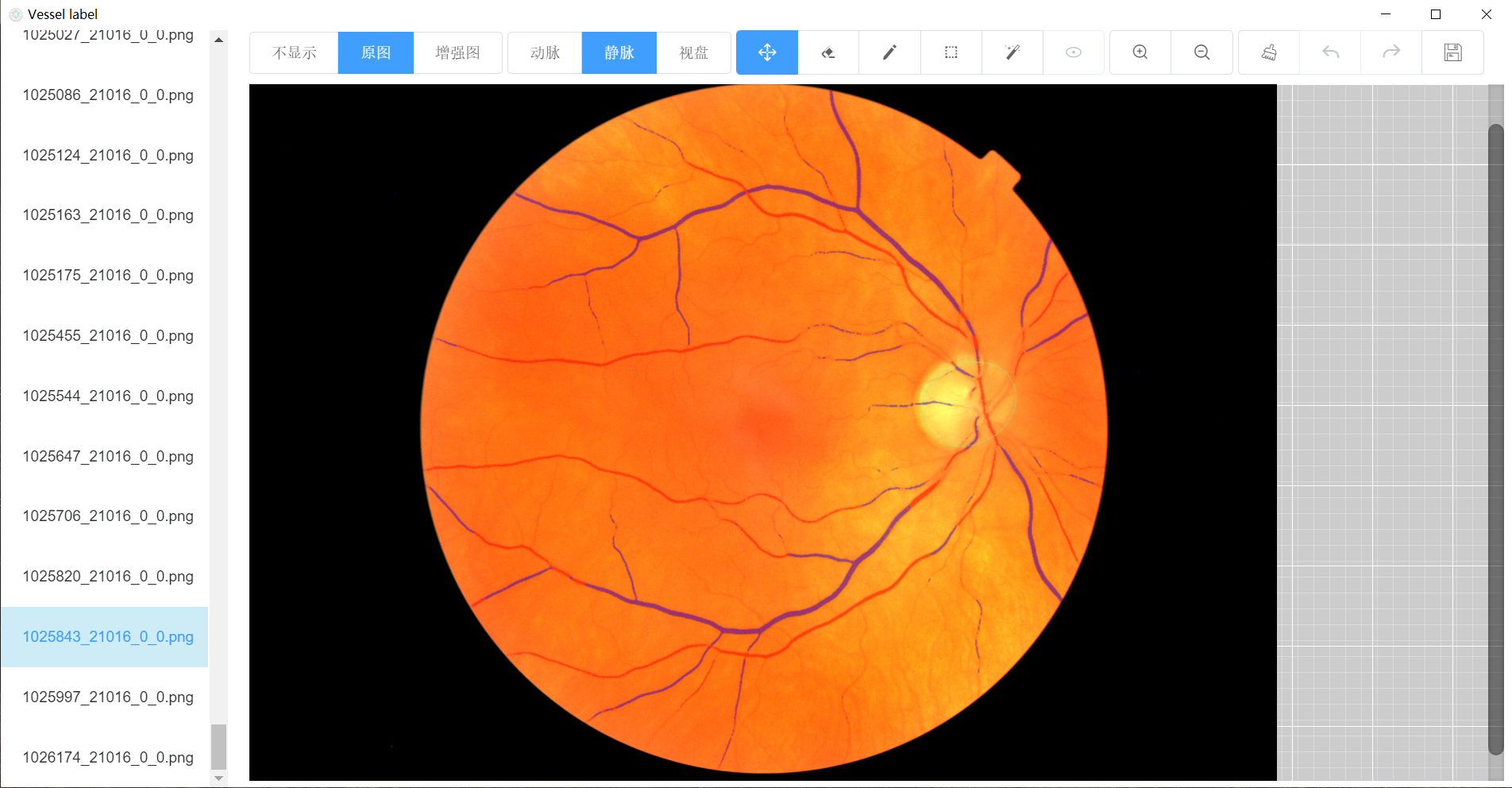


Figure 2. Diagram demonstrating the train/test split


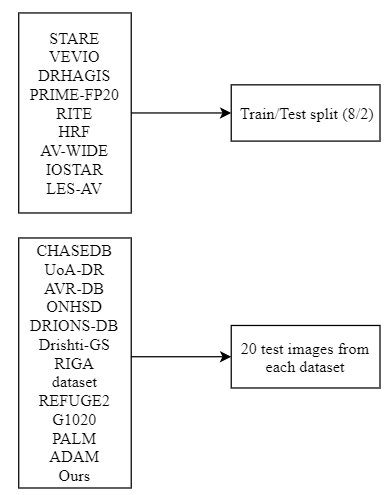


**Figure 3.** Manual measures in the REVIEW database versus Retina-based Microvascular Health Assessment System (RMHAS) prediction. A. CLRIS (Central Light Reflex Image Set), B. HRIS (High Resolution Image Set), C.VDIS (Vascular Disease Image Set)


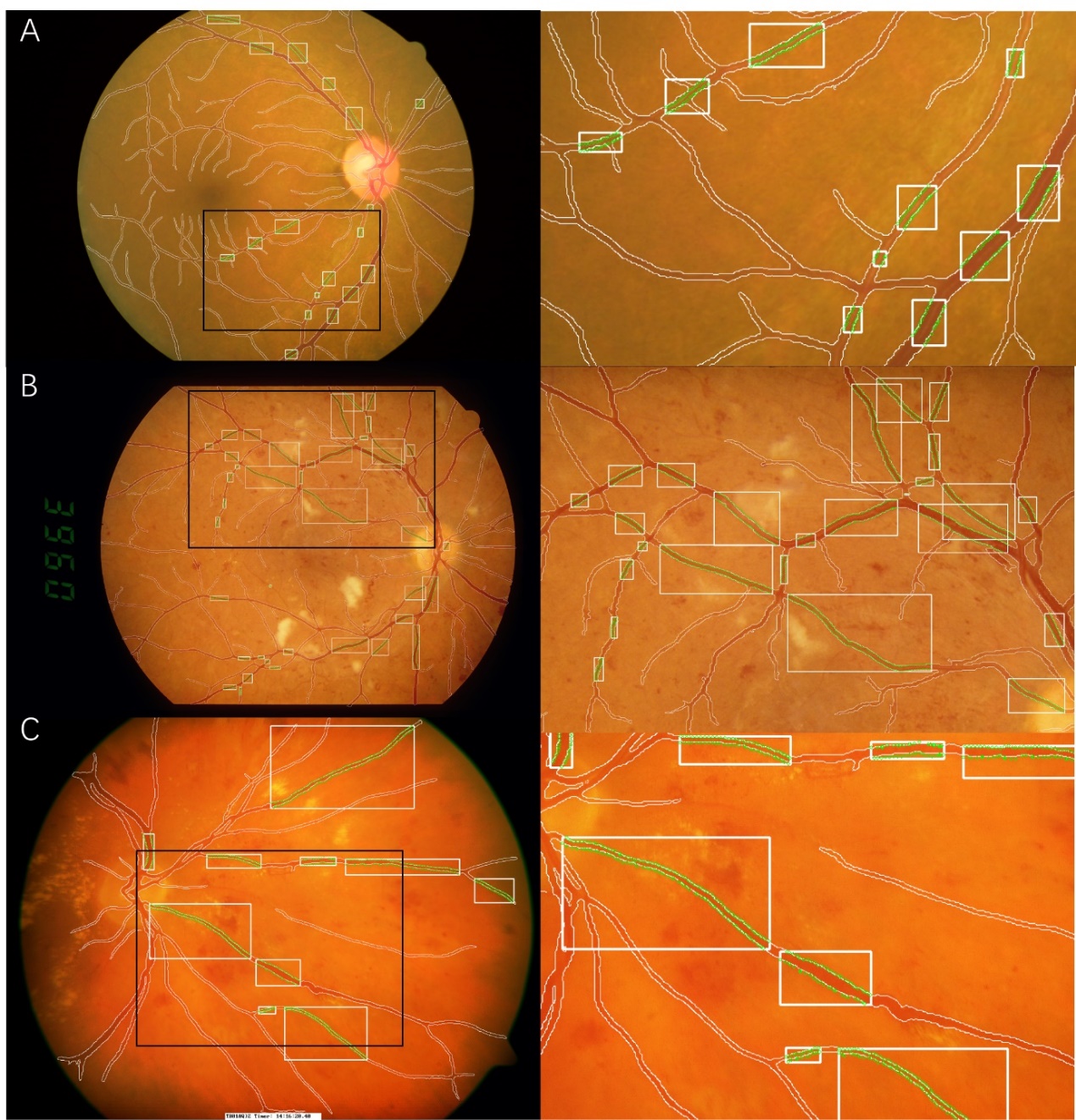


**Figure 4.** Bland-Altman plots show the agreement between measured parameters of Retina-based Microvascular Health Assessment System (RMHAS) predictions and manual segmentations. (A) retinal arteriolar caliber; (B) retinal venular caliber; (C) AVRe between manual and predicted vessel maps; (D) differences between AVRe measures on manual and predict vessel maps; versus the distance of the optic disc center to the edge of FOV. CRAE, central retinal artery equivalent; CRVE, central retinal vein equivalent. AVRe, artery to vein ratio obtained from vessel equivalents; FOV, field of view. CRAE, CRVE were in pixel units.

A B


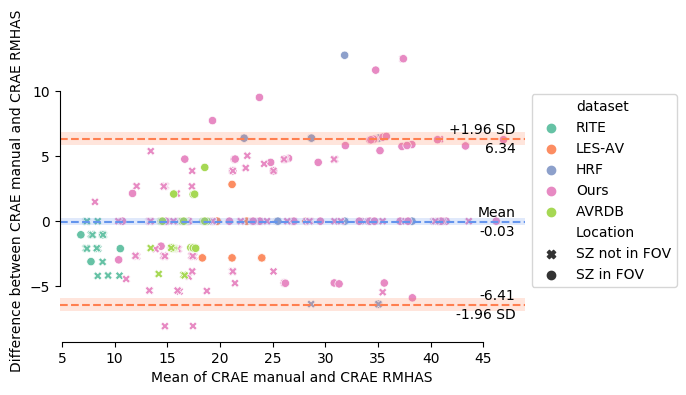

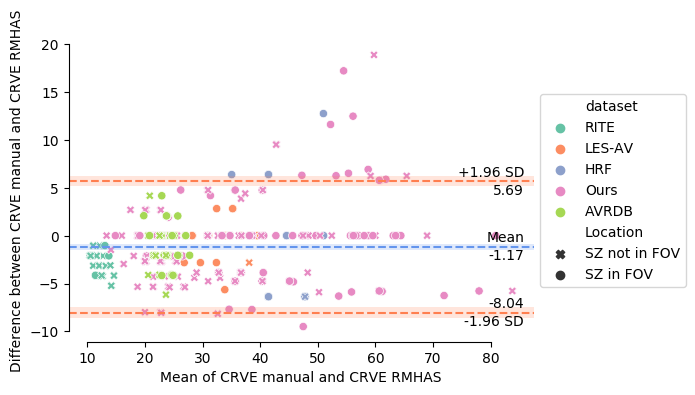


C D


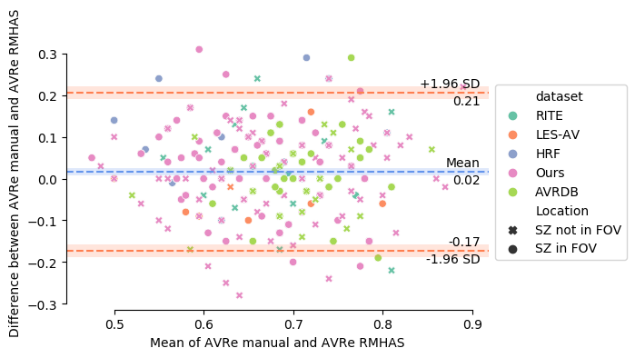

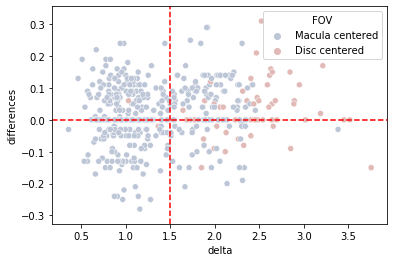

Supplement: Supplementary file 1 [file Data_Sheet_1.docx]
